# A prospective study comparing patient-reported outcomes in Crohn’s disease

**DOI:** 10.1097/MEG.0000000000001568

**Published:** 2019-12-09

**Authors:** Daniël R. Hoekman, Mark Löwenberg, Gijs R. van den Brink, Cyriel Y. Ponsioen, Marc A. Benninga, Geert R. D’Haens

**Affiliations:** Departments of a Gastroenterology and Hepatology; b Pediatric Gastroenterology and Nutrition, Amsterdam University Medical Center, Amsterdam, The Netherlands

**Keywords:** Bristol stool form scale, Bristol stool chart, Crohn’s disease, patient-reported outcomes, visual analog scale

## Abstract

Patient reported outcomes are important in Crohn’s disease. In this prospective cohort, we investigated the performance of the Bristol Stool Form Scale (BSFS) and a visual analog scale (VAS) for abdominal pain as outcome measures in Crohn’s disease.

**Methods:**

Patients with active Crohn’s disease starting glucocorticoids or anti-tumor necrosis factor were included. Before treatment and 10 weeks later we collected: clinical activity [Harvey Bradshaw Index (HBI) and Crohn’s-Disease-Activity-Index (CDAI)], serum C-reactive protein (CRP) and fecal calprotectin, and BSFS (1–7) and a 100-mm VAS based on a 7-day diary. Clinical response was defined as a reduction by at least 3 and at least 100 of HBI and CDAI, respectively. Fecal calprotectin-response and CRP-response were defined as reduction of at least 50%.

**Results:**

Thirty-eight patients completed follow-up. At baseline, BSFS-parameters correlated more strongly with clinical activity (range: *r*_*s*_: 0.31–0.74) than with CRP (*r*_*s*_: −0.01 to 0.16) and fecal calprotectin (*r*_*s*_: 0.14–0.26). VAS scores correlated very weakly to moderately with clinical activity (*r*_*s*_: 0.18–0.45), and weakly to moderately with CRP (*r*_*s*_: 0.24–0.34) and fecal calprotectin (*r*_*s*_: 0.35–0.43). Changes in VAS scores correlated moderately to strongly (*r*_*s*_: 0.55–0.71) with changes in clinical activity, and weakly with changes in CRP and fecal calprotectin (*r*_*s*_: 0.21–0.35). Changes in BSFS parameters correlated weakly to moderately (*r*_*s*_: 0.23–0.53) with changes in clinical activity, and very weakly to weakly (*r*_*s*_: 0.01–0.35) with changes in CRP and fecal calprotectin. Responsiveness of VAS and BSFS was moderate to high (Guyatt’s statistic 0.41–2.17) and highly dependent on the definition of response.

**Conclusions:**

The BSFS and a VAS appear to be responsive with moderate-to-strong construct validity to monitor patients with Crohn’s disease.

## Abstract 2

Supplemental Digital Content is available in the text.

## Introduction

Traditionally, the efficacy of therapeutic interventions in clinical trials in Crohn’s disease was assessed using the Crohn’s Disease Activity Index (CDAI), an index consisting of laboratory tests, physical examination and patient-reported symptoms based on a 7-day diary [1]. Although the CDAI correlates well with the physician’s overall global assessment of disease activity [1], it is not disease specific and it correlates very poorly with endoscopic severity [2], an important predictor of long-term outcome [3]. Consequently, it has been suggested that mucosal healing in combination with patient-reported outcomes (PROs) should be a primary outcome measure in clinical trials [4,5].

Because the CDAI includes components that are not reported by patients (abdominal mass assessed by a physician, body weight, hematocrit), it does not qualify as a genuine PRO. Furthermore, the symptom-related items in the CDAI were selected based on the gastroenterologist’s point-of-view [1], rather than on what is important for the patient, and the items’ weights were arbitrarily chosen rather than based on rigorous biometric techniques.

PROs can be defined as any report of the status of a patient’s health condition that comes directly from the patient without interpretation of the patient’s response by a clinician or anyone else [6], and are increasingly recommended to be used as outcome measures in clinical trials and daily practice. Recently, the United States Food and Drug Administration (FDA) proposed guidance for the development of PROs [6]. According to this guidance, the CDAI is no longer acceptable as a primary outcome in clinical trials [6].

However, the development of PROs according to the FDA guidance is a lengthy process involving patient concept elicitation interviews, expert interviews, item generation, content validity testing, patient cognitive interviews and quantitative validation [7]. Currently, no PRO instrument is available for Crohn’s disease which is developed in accordance with the FDA guidance.

In clinical practice and in trials, the Bristol Stool Form Scale (BSFS) and a visual analogue scale (VAS) are frequently used to evaluate bowel habits and symptoms of abdominal pain and discomfort. To our knowledge, these instruments have never been evaluated as outcome measures in Crohn’s disease, although they are truly ‘patient reported’. We hypothesized that the BSFS and VAS scores for abdominal pain correlate well with clinical and biochemical disease activity, and that these measures could be used as outcome measures in Crohn’s disease. In this prospective study, we investigated whether the BSFS and a VAS for abdominal pain can be used as outcome measures in Crohn’s disease. Therefore, we examined the criterion validity and responsiveness of these instruments in patients with Crohn’s disease who start tumor necrosis factor (TNF) inhibitors or corticosteroid treatment (treatments of known efficacy) for active disease.

## Methods

### Population

We performed a single center, prospective, observational study at the Academic Medical Center in Amsterdam, The Netherlands, between October 2013 and February 2016. Eligible patients were 18 years of age or older with an endoscopically and histologically confirmed diagnosis of Crohn’s disease, scheduled to start oral corticosteroids or TNF inhibitors for active disease based on gastrointestinal symptoms in combination with biochemical evidence of inflammation [high-sensitivity serum C-reactive protein (CRP) > 5 mg/L and/or fecal calprotectin > 250 µg/g]. Patients with an ostomy or a history of colectomy were excluded. Oral prednisolone was administered at a daily dose of 40 mg for 3 weeks (irrespective of body weight), followed by a gradual tapering by 5 mg every week in the case of clinical response. Budesonide was administered orally at a daily dose of 9 mg for 8 weeks, followed by a 2-week period at a daily dose of 6 mg. Infliximab was administered intravenously at a dose of 5 mg per kg bodyweight at week 0, 2 and 6. Adalimumab was administered subcutaneously at 160 mg at week 0, 80 mg at week 2, followed by 40 mg every other week.

### Outcome measures

Comprehensive measurement of disease activity and PROs was performed prior to treatment and 10 weeks later. Study outcomes that were studied were divided in three categories: (1) the ‘conventional’ diseases activity scores: CDAI and Harvey Bradshaw Index (HBI); (2) biochemical markers of disease activity: high sensitivity serum CRP and fecal calprotectin (Bühlmann ELISA, Schönenbuch, Switzerland); and (3) PROs: BSFS, a visual analogue abdominal pain scale, PRO2 and PRO3 (see below).

During the week prior to treatment and 10 weeks after the initiation of treatment, participants were asked to complete a diary (provided in supplements, Supplemental digital content 1, http://links.lww.com/EJGH/A464) for seven consecutive days for daily recording of the number of loose/watery stools, abdominal pain, general well-being (the three components of the CDAI), abdominal pain on a 100 mm VAS, and for the daily scoring of the appearance of the feces: patients were asked to score which types of stools they have had on the BSFS (1–7). HBI scores were recorded on the day of the visit, reflecting symptoms on the day prior to the visit. The PRO measures PRO2 and PRO3 were calculated from CDAI diaries, as described previously. For each diary component of the CDAI, the daily average score over 7 days was weighted using the original CDAI multiplication factors. Subsequently, the scores for all items (number of loose/watery stools, abdominal pain, ± general well-being) were summed to create PRO2 and PRO3, respectively [8].

Clinical response was based on HBI and CDAI scores. HBI response was defined as a reduction in HBI by ≥3 points [9]. CDAI response was defined as a reduction in CDAI ≥100 points, as frequently used in clinical trials [10]. Clinical remission was defined as a CDAI score of <150 and/or an HBI score of ≤4. Biochemical response was defined as a reduction of ≥50% compared with baseline values of fecal calprotectin (fecal calprotectin response) and/or CRP (CRP response), as used previously [11]. Diarrhea on the BSFS was defined as a score of ≥6. Severe diarrhea was defined as a BSFS score of 7. Constipation was defined as a BSFS score of ≤2 [12].

### Sample size

The primary aim of the study was to evaluate the correlation between changes in BSFS and VAS score with changes in clinical and biochemical outcomes as a measure of criterion validity. A sample of 36 patients is generally deemed sufficient to detect an anticipated correlation coefficient of 0.45 different from 0.00 with 80% power at the 0.05 (two-sided) level of significance.

### Statistical analysis

At baseline, Spearman’s rank correlation of the following outcomes with clinical scores (HBI, CDAI, PRO2 and PRO3) and biochemical outcomes (CRP, fecal calprotectin) were studied: mean VAS pain score over 1 week, VAS score at day 1 of the diary (D1), proportion of days without diarrhea based on the BSFS, proportion of days without severe diarrhea based on the BSFS, the highest BSFS score at D1 and the highest BSFS score during the whole week. Furthermore, Spearman’s rank correlation of the following outcomes was evaluated: change in mean VAS score over 1 week, VAS score at D1, proportion of days without diarrhea based on the BSFS, proportion of days without severe diarrhea based on the BSFS, highest BSFS score at D1 and highest BSFS score over 1 week. Mean BSFS scores were not analyzed, because they can be misleading [e.g., the mean score of constipation (BSFS = 1) and liquid stools (BSFS = 7) are normal stool (BSFS = 4)]. Correlation coefficients were interpreted as follows: 0–0.2 very weak; 0.2–0.4 weak; 0.4–0.6 moderate; 0.6–0.8 strong; 0.8–1.0 very strong [13]. Guyatt’s responsiveness statistic was calculated as the mean change in responders divided by the SD of the change in nonresponders. Values of 0.2–0.5, 0.5–0.8 and ≥0.8 were considered to represent low, moderate and high responsiveness, respectively [14].

### Missing data

In the case of missing days in the diary, data from the completed days were analyzed. If more than 4 days of a diary were missing, patients were excluded from the analysis. In the case of missing CRP and/or fecal calprotectin levels, patients were excluded from the corresponding analyses only. In the case of missing hematocrit levels, the CDAI score was calculated based on the last observation carried forward principle using the last recorded hematocrit level.

## Ethical considerations

The study protocol was reviewed by the local ethics committee, who deemed that, in line with the Dutch Medical Research Involving Human Subjects Act (Wet Medisch-Wetenschappelijk Onderzoek met Mensen), full ethical review was not required.

## Results

### Study population

Fifty-one patients participated in the study, of whom 38 completed the study and were included in these analyses. Baseline characteristics are provided in Table 1. Reasons for not completing the study were noncompliance with diaries (*n* = 7), withdrawal of consent (*n* = 3), loss to follow-up (*n* = 2) and a revised diagnosis (no Crohn’s disease; *n* = 1). The median CDAI score at baseline was 173 points interquartile range [(IQR) 125–225]. Median CRP and fecal calprotectin levels were 4.9 mg/L (IQR 2.4–15.2) and 825 µg/g (291–1800), respectively.

**Table 1.**
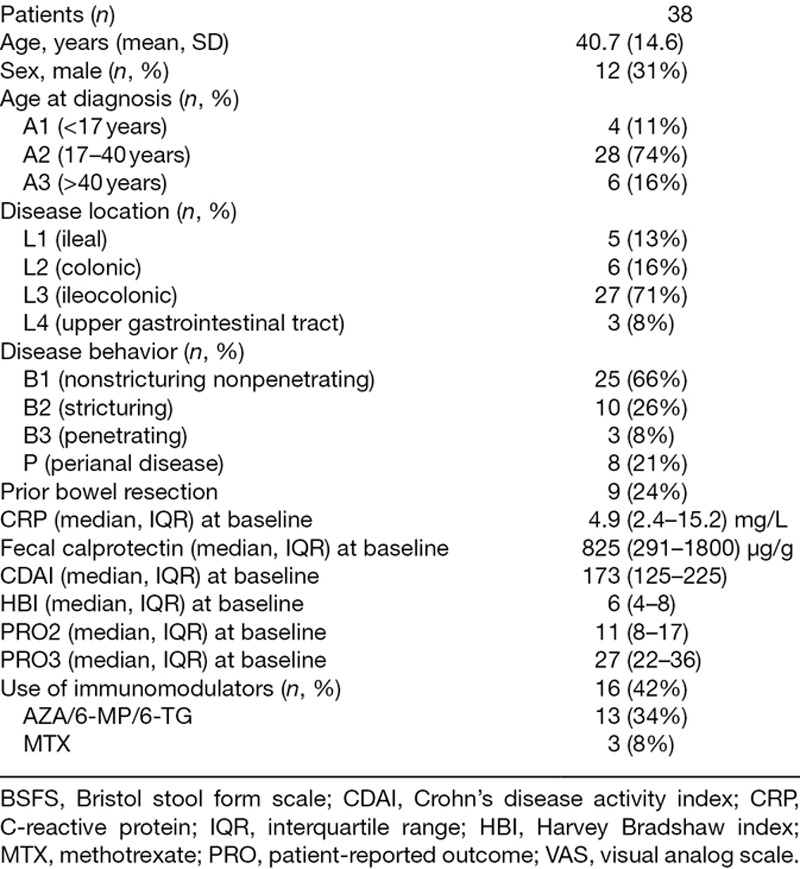
Baseline characteristics

### Treatment efficacy

Corticosteroids were started in 24 (63%) patients [7 (18%) prednisolone, 17 (45%) budesonide], and TNF inhibitors in 14 (37%) patients [6 (16%) infliximab, 8 (21%) adalimumab]. Clinical response at week 10 based on HBI and CDAI was observed in 14 (37%) and 8 (21%) patients, respectively, while CRP and fecal calprotectin response was seen in 24 (63%) and 16 (47%) patients, respectively. The proportion of patients in remission at week 10 was 63%, based on either HBI or CDAI.

### Bristol Stool Form Scale

#### Descriptive statistics

**Fig. 1.**
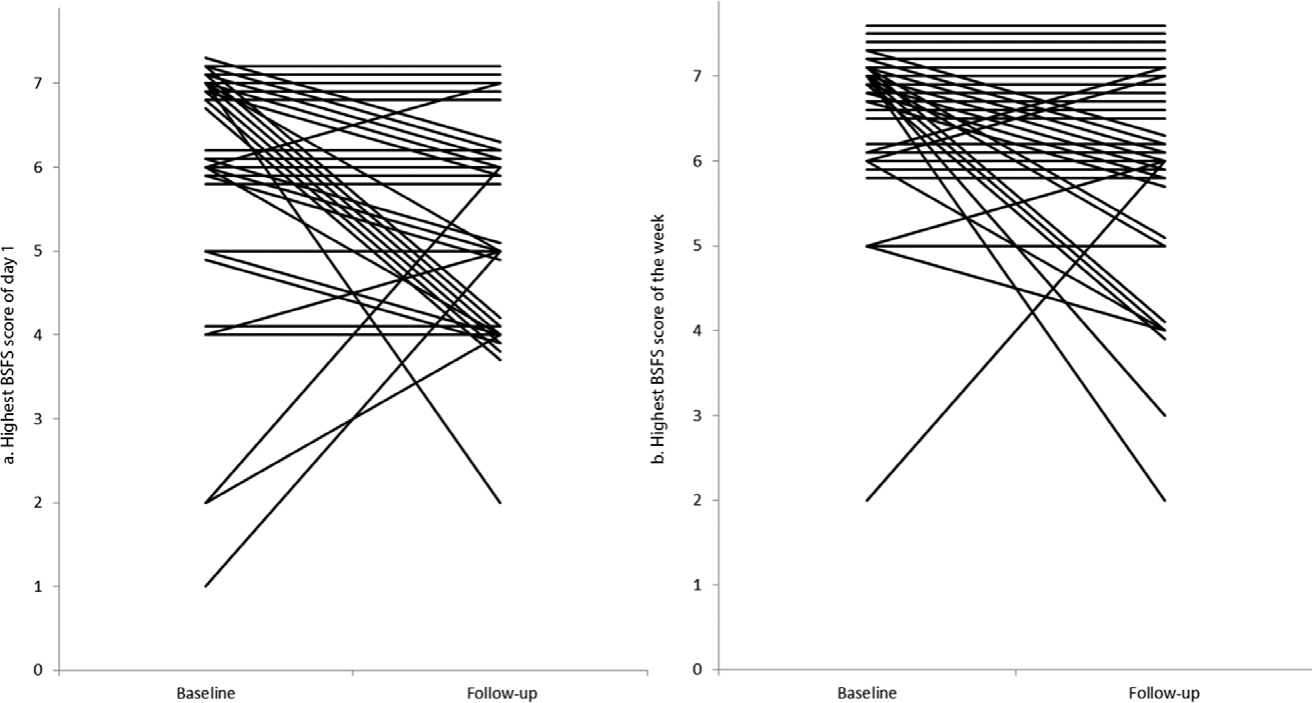
Highest BSFS score of (a) day 1 and (b) week 1 before and after treatment. Lines represent individual patients. All values represent absolute numbers. Lines have been moved vertically to allow for the visualization of overlapping data. BSFS, Bristol stool form scale.

Maximum BSFS scores at baseline and after treatment are provided in Fig. 1. An overview of all reported BSFS scores is provided in Supplementary Fig. 1, Supplemental digital content 2, http://links.lww.com/EJGH/A465. At baseline, the majority of patients had diarrhea and severe diarrhea at day 1 (*n* = 29, 76%; *n* = 19, 50%, respectively), or at least one day during the week (*n* = 34, 89%; *n* = 26, 68%, respectively). After treatment, 28 (74%) patients still suffered from diarrhea at least one day during the week, and 14 (37%) patients had severe diarrhea at least once during the week.

Prior to treatment, 6 (16%) patients had constipation-type stools at day 1, and 10 (26%) patients had constipation-type stools at least one day during the week. This decreased to 2 (5%) and 8 (21%) after treatment, respectively.

#### Relationship with disease activity

The correlation of BSFS scores at baseline with clinical disease activity index scores, PRO2, PRO3, fecal calprotectin and CRP is provided in Table 2. The highest BSFS score correlated weakly to moderately with clinical disease activity (*r*_*s*_: 0.31–0.54) and weakly to very weakly with CRP (*r*_*s*_: −0.01 to 0.16) and fecal calprotectin (*r*_*s*_: 0.14–0.27). The proportion of days with diarrhea or severe diarrhea correlated best with CDAI (*r*_*s*_: 0.42–0.50) and HBI (*r*_*s*_: 0.51–0.69). BSFS outcomes correlated more strongly with PRO2 score (*r*_*s*_: 0.54–0.74) compared to PRO3 score (*r*_*s*_: 0.29–0.53).

**Table 2.**
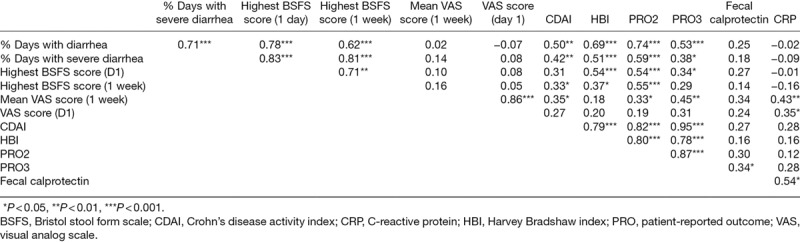
Spearman’s rank correlation coefficients of Bristol stool form scale score, visual analog scale score, Crohn’s disease activity index, Harvey Bradshaw index, patient-reported outcome 2, patient-reported outcome 3, fecal calprotectin and C-reactive protein at baseline

#### Criterion validity and responsiveness to change

**Fig. 2.**
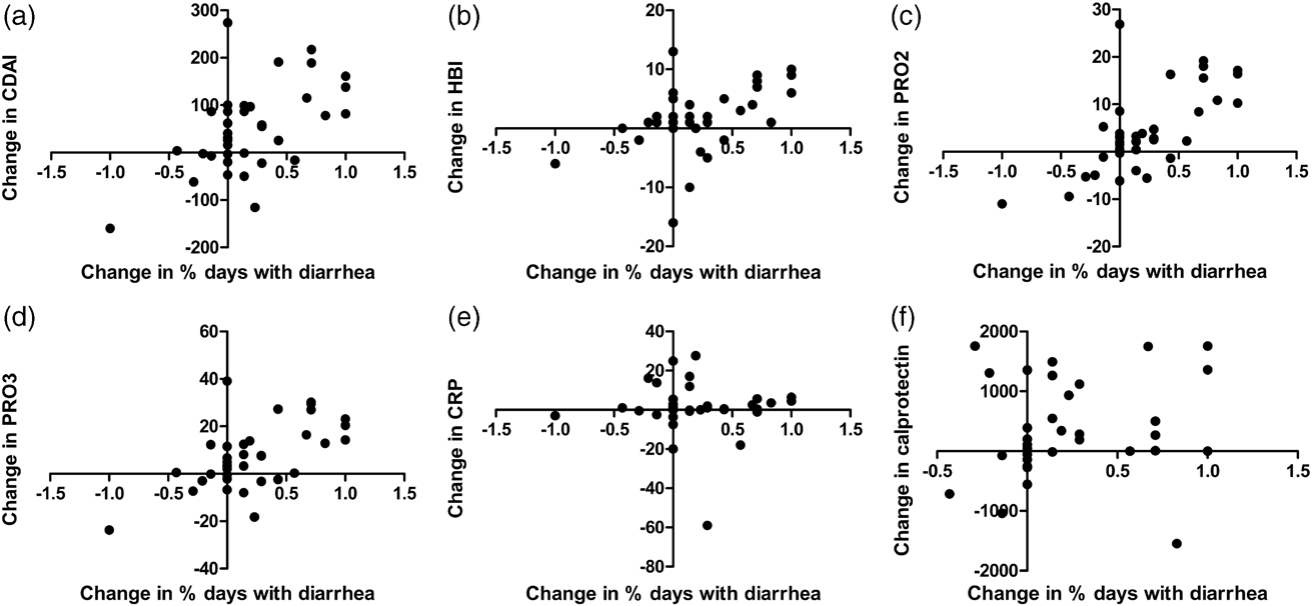
Correlation between changes in the proportion of days with diarrhea based on BSFS and changes in CDAI (a), HBI (b), PRO2 (c), PRO3 (d), fecal calprotectin (e) and CRP (f). Spearman’s rank correlation coefficients are provided (*r*_*s*_). BSFS, Bristol stool form scale; CDAI, Crohn’s disease activity index; CRP, C-reactive protein; HBI, Harvey Bradshaw index; PRO, patient-reported outcome.

The correlation between changes in BSFS scores and changes in clinical disease activity index scores, PRO2, PRO3, fecal calprotectin and CRP after treatment compared with baseline is provided in Table 3 and Fig. 2a–f. Changes in BSFS outcomes correlated weakly to moderately to changes in clinical disease activity indices (*r*_*s*_: 0.23–0.53), and weakly to very weakly with changes in fecal calprotectin (*r*_*s*_: 0.10–0.35) and CRP (*r*_*s*_: 0.01–0.15). At week 10, similar to baseline, changes in BSFS outcomes correlated more strongly with changes in PRO2 score (*r*_*s*_: 0.47–0.63) compared with changes in PRO3 score (*r*_*s*_: 0.27–0.56).

**Table 3.**
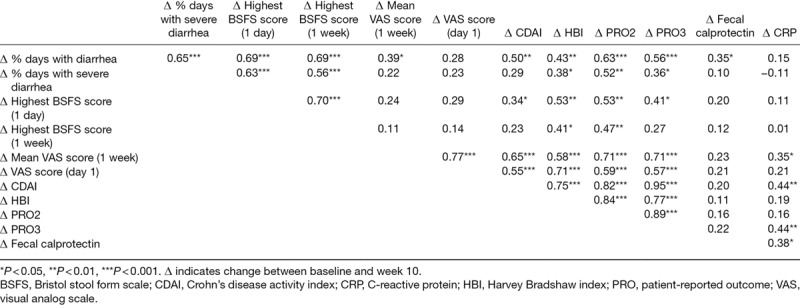
Spearman’s rank correlation coefficients of changes in Bristol stool form scale score, visual analog scale score, Crohn’s disease activity index, Harvey Bradshaw index, patient-reported outcome 2, patient-reported outcome 3, fecal calprotectin and C-reactive protein between baseline and after treatment

Guyatt’s responsiveness statistics of changes in proportion of days with diarrhea with respect to response based on CDAI and HBI were respectively 1.82 and 1.48, indicating high responsiveness and 0.68 and 0.70 for response based on fecal calprotectin and CRP, respectively, indicating moderate responsiveness. Guyatt’s statistics of changes in proportion of days with severe diarrhea were 1.57, 1.12, 0.41 and 0.28, respectively. Guyatt’s statistics of highest BSFS score during the whole week were 1.10, 1.40, 0.60 and 0.57, respectively. Guyatt’s statistics of highest BSFS score at day 1 were 1.39, 1.36, 0.61 and 0.69, respectively.

### Visual analog scale for abdominal pain

#### Descriptive statistics

At baseline, the mean VAS score at day 1 was 36 mm (SD: 23), and mean average VAS score during the week was 34 mm (SD: 21). After treatment, the mean VAS score on the first day of the diary was 24 mm (SD: 27), and mean average VAS score during week of the diary was 20 mm (SD: 20).

#### Relationship with disease activity

The correlation of VAS scores at baseline with the clinical disease activity index scores, PRO2, PRO3, fecal calprotectin and CRP is shown in Table 2. At baseline, the VAS score correlated weakly to very weakly with clinical disease activity (*r*_*s*_: 0.18–0.35) and PRO2 (*r*_*s*_: 0.19–0.33) and weakly to moderately with biochemical disease activity outcomes (*r*_*s*_: 0.24–0.43) and PRO3 (*r*_*s*_: 0.31–0.45).

#### Criterion validity and responsiveness to change

**Fig. 3.**
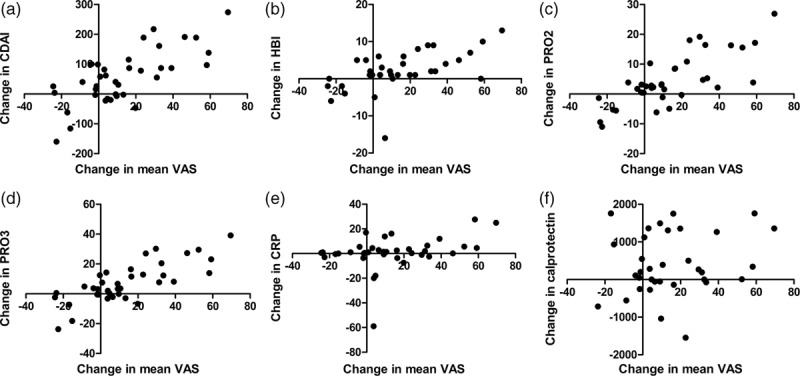
Correlation between changes in mean VAS score and changes in CDAI (a), HBI (b), PRO2 (c), PRO3 (d), fecal calprotectin (e) and CRP (f). Spearman’s rank correlation coefficients are provided (*r*_*s*_). BSFS, Bristol stool form scale; CDAI, Crohn’s disease activity index; CRP, C-reactive protein; HBI, Harvey Bradshaw index; PRO, patient-reported outcome; VAS, visual analog scale.

The correlations between changes in VAS scores and changes in clinical disease activity index scores, fecal calprotectin and CRP after treatment are provided given in Table 3 and Fig. 3a–f. Changes in VAS scores correlated moderately to strongly with changes in clinical disease activity (*r*_*s*_: 0.55–0.71), PRO2 (*r*_*s*_: 0.59–0.71) and PRO3 (*r*_*s*_: 0.57–0.71), whereas only a weak correlation was observed with changes in fecal calprotectin (*r*_*s*_: 0.21–0.23) and CRP (*r*_*s*_: 0.21–0.35).

Guyatt’s responsiveness statistics of mean VAS score for response based on CDAI and HBI, fecal calprotectin and CRP were 2.17, 1.38, 1.30 and 1.11, respectively, indicating high responsiveness. Guyatt’s statistics of VAS score at D1 were 1.59, 1.12 (high responsiveness), 0.76 and 0.62 (moderate responsiveness), respectively.

## Discussion

In this prospective observational study, we evaluated the criterion validity and responsiveness of the BSFS and VAS as outcome measures for Crohn’s disease in patients starting with corticosteroids or TNF inhibitors for active disease. BSFS showed a weak-to-strong correlation with various clinical disease activity indices at baseline and changes in BSFS correlated weakly to moderately with changes in clinical disease activity after treatment. VAS score (for abdominal pain) correlated weakly with clinical disease activity at baseline, while a moderate-to-strong correlation of changes in VAS score was observed with changes in clinical disease activity after treatment. Biochemical disease activity correlated weakly to very weakly with BSFS and VAS scores, both at baseline and when evaluated as change after treatment compared to baseline. Responsiveness of both BSFS and VAS was large with respect to clinical response, and moderate with respect to biochemical response.

Overall, both BSFS and VAS were more strongly related to clinical disease activity indices compared to biochemical markers. This was to be expected, considering that in both clinical activity indices abdominal pain and liquid or soft stools are important components of the total score [1,15]. For clinical practice and trials, it may therefore be preferable to score stool frequency and abdominal pain separately, with different responsiveness criteria. In reality, Crohn’s disease patients with colonic inflammation have predominantly diarrhea, whereas patients with small bowel disease tend to suffer more from abdominal pain and less diarrhea. It would be interesting to separate patients with colonic and small bowel disease. Unfortunately, subgroups were too small for meaningful analyses.

Generally, changes in VAS score were more closely related to changes in both clinical and biochemical disease activity, compared to BSFS outcomes. This may be related to the fact that symptoms of diarrhea often persist in patients with Crohn’s disease. Indeed, in our cohort, the vast majority of patients (74%) still suffered from diarrhea at least weekly. Considering that biochemical response was observed in roughly half of our patients, part of these symptoms of diarrhea may be explained by coexisting diarrhea-predominant irritable bowel syndrome and by bile acid diarrhea, both of which are known to occur frequently in patients with Crohn’s disease [16,17].

For the BSFS and VAS, there appeared to be little advantage to determine the highest score over a seven-day period compared to using the score on the first day of the week. This may increase the feasibility and usability in both trials and clinical practice where the BSFS and VAS scores are of interest. However, the proportion of days with diarrhea derived from the diary correlated most strongly with disease activity and was found to be most responsive.

Strength of this study is the prospective collection of data in a real-life cohort of patients with Crohn’s disease who require TNF inhibitors or corticosteroids for active disease. There are, however, some limitations. First, endoscopy was neither performed at inclusion nor during follow-up. Hence, biological response was determined using the surrogate markers CRP and fecal calprotectin rather than endoscopy. Furthermore, patients were asked daily to score which types of stools they have had on the BSFS, but not the relative frequency of each individual BSFS stool type. Consequently, it was not possible to distinguish between patients who had soft or liquid stools only once during the day and those with more severe symptoms of diarrhea. Another potential limitation is that compliance with paper diaries may be low and backfilling of diaries based on recall may influence outcomes [18].

Although this study provides evidence that BSFS and VAS for abdominal pain may be used as outcome measures in patients with Crohn’s disease, our results are insufficient to recommend the routine use of these instruments in clinical trials or in clinical practice. Prior to routine implementation, our findings should first be validated in an independent cohort. Based on our study, ideal BSFS and VAS candidate outcomes are (1) the proportion of days with diarrhea (i.e., BSFS ≥6) during 1 week, (2) one-day highest BSFS score, and (3) one-day VAS abdominal pain score.

### Conclusion

The BSFS and a VAS for abdominal pain appear to be responsive instruments with moderate to strong construct validity to monitor patients with Crohn’s disease. Future studies are required to validate these findings in an independent cohort.

## Acknowledgements

We thank Larry Stitt for his valuable comments on the manuscript.

## Conflicts of interest

G.D.H. has served as advisor for Abbvie, Ablynx, Actogenix, Amakem, Amgen, AM Pharma, AstraZeneca, Avaxia, Bristol Meiers Squibb, Boerhinger Ingelheim, Celgene, Celltrion, Cosmo, Covidien, Elan, Ferring, DrFALK Pharma, Centocor/Jansen Biologics, Engene, Ferring, Galapagos, Gilead, Glaxo Smith Kline, Hospira, Medimetrics, Millenium/Takeda, Mitsubishi Pharma, Merck Sharp Dome, Mundipharma, Novonordisk, Otsuka, Pfizer, Protein Design Laboratories, Prometheus laboratories/Nestle, Receptos, Robarts Clinical Trials, Salix, Sandoz, Setpoint, Shire, Teva, Tigenix, Tillotts, Topivert, UCB, Versant and Vifor and received speaker fees from Abbvie, Ferring, Jansen Biologics, Merck Sharp Dome, Mundipharma, Norgine, Shire, Takeda, Tillotts, UCB and Vifor. C.Y.P. received grant support from Takeda, speaker’s fee from Takeda, Abbvie, and Dr Falk Pharma, and consultancy fee from Takeda. M.L. received speaker fees from Abbvie, Celgene, Covidien, Dr. Falk, Ferring Pharmaceuticals, Gilead, GlaxoSmithKline, Janssen-Cilag, Merck Sharp & Dohme, Pfizer, Protagonist therapeutics, Receptos, Takeda, Tillotts and Tramedico, and has received grant support from AbbVie, Merck Sharp & Dohme, Achmea healthcare and ZonMW. G.B. is an employee of GlaxoSmithKline. There are no conflicts of interest for the remaining authors.

## Supplementary Material
