# Supplementary material for: A prospective study comparing patient-reported outcomes in Crohn’s disease
Source: Eur J Gastroenterol Hepatol. 2019 Dec 9;32(1):38–44. doi: 10.1097/MEG.0000000000001568 (PMC6903328; doi:10.1097/MEG.0000000000001568)
Supplement: Supplementary file 1 [file ejgh-32-38-s001.pdf]

EXPLANATION :

Please complete this diary to keep track of your abdominal complaints and bowel habits.

We ask you to complete several questions for 7 consecutive days. Although some questions may appear to be very similar, please complete all questions. Below is a brief explanation.

Using the following line, we ask you to indicate whether or not you had abdominal pain, and to indicate the severity of the abdominal pain.

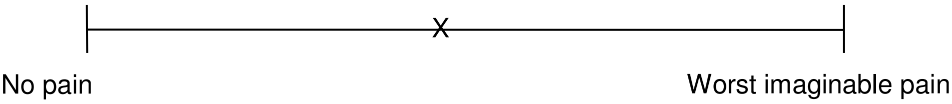

Using the following images, please keep track of the appeareance of your stool.

**1**	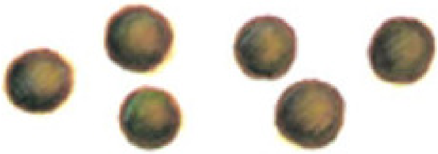	**Seperate hard lumps, like nuts (hard to pass)**
**2**	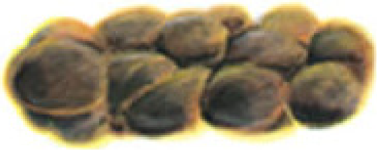	**Sausage-shaped but lumpy**
**3**	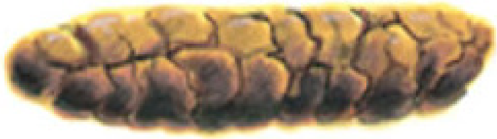	**Like a sausage but with cracks on the surface**
**4**	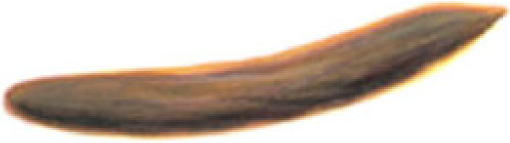	**Like a sausage or snake, smooth and soft**
**5**	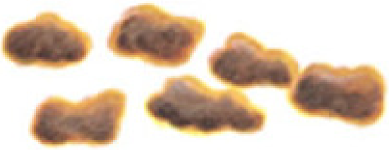	**Soft blobs with clear-cut edges**
**6**	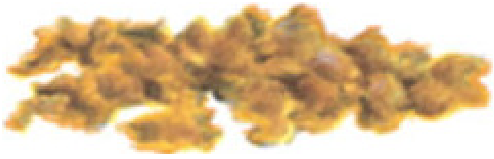	**Fluffy pieces with ragged edges, a mushy stool**
**7**	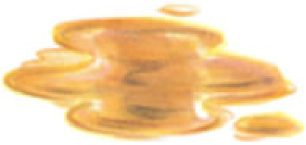	**Watery, no solid pieces.<br>Entirely liquid.**

**Day ( )**	|__|__| / |__|__| / |2|0|1|__| day month year

**Please mark on the line with an 'x' the degree of abdominal pain you've had today**<br>No pain ——————— Worst imaginable pain	
**Abdominal pain**	O None<br>O Mild<br>O Moderate<br>O Severe
**How many stools did you pass today?**	|__|__|__| stools
**What was the appearance of the stools?**<br>(please mark all the different shapes you've had)<br><br>Based on the images on the explanation page.	☐ 1 ☐ 5<br>☐ 2 ☐ 6<br>☐ 3 ☐ 7<br>☐ 4
**How often did you have very soft or liquid stools today?**	|__|__|__|
**General well-being**	O Well<br>O Slightly below par<br>O Poor<br>O Very poor<br>O Terrible
**Taking anti-diarrhoeal drugs**	O Yes, specify:<br>____________________<br><br>O No
**Fever above 37.8 °C**	O Yes, specify temperature:<br>_____ °C<br><br>O No
