# Supplementary material for: A prospective study comparing patient-reported outcomes in Crohn’s disease
Source: Eur J Gastroenterol Hepatol. 2019 Dec 9;32(1):38–44. doi: 10.1097/MEG.0000000000001568 (PMC6903328; doi:10.1097/MEG.0000000000001568)

Supplemental figure 1. Overview of BSFS scores at baseline and after treatment. A decrease in diarrhea and severe diarrhea was observed after treatment.

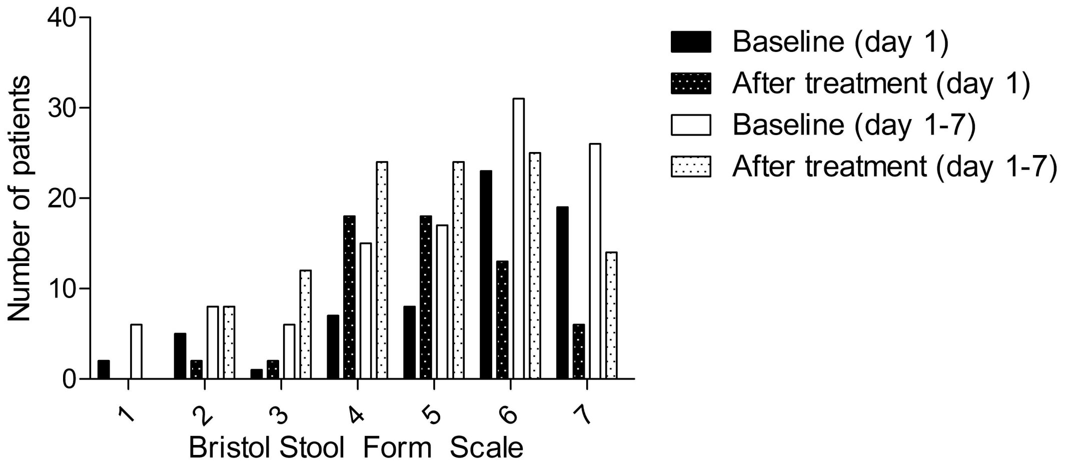

Supplement: Supplementary file 2 [file ejgh-32-38-s002.pdf]
